# Key brain cell interactions and contributions to the pathogenesis of Alzheimer’s disease

**DOI:** 10.3389/fcell.2022.1036123

**Published:** 2022-11-29

**Authors:** Joana Saavedra, Mariana Nascimento, Márcia A. Liz, Isabel Cardoso

**Affiliations:** ^1^ Instituto de Investigação e Inovação em Saúde (i3S), Universidade do Porto, Porto, Portugal; ^2^ Instituto de Biologia Molecular e Celular (IBMC), Universidade do Porto, Porto, Portugal; ^3^ Instituto de Ciências Biomédicas Abel Salazar (ICBAS), Universidade do Porto, Porto, Portugal; ^4^ Faculdade de Medicina, Universidade do Porto, Porto, Portugal

**Keywords:** Alzheimer’s disease, neural integrity, cell-cell signaling, neuroinflammation, neurodegeneration

## Abstract

Alzheimer’s disease (AD) is the most common neurodegenerative disease worldwide, with the two major hallmarks being the deposition of extracellular β-amyloid (Aβ) plaques and of intracellular neurofibrillary tangles (NFTs). Additionally, early pathological events such as cerebrovascular alterations, a compromised blood-brain barrier (BBB) integrity, neuroinflammation and synaptic dysfunction, culminate in neuron loss and cognitive deficits. AD symptoms reflect a loss of neuronal circuit integrity in the brain; however, neurons do not operate in isolation. An exclusively neurocentric approach is insufficient to understand this disease, and the contribution of other brain cells including astrocytes, microglia, and vascular cells must be integrated in the context. The delicate balance of interactions between these cells, required for healthy brain function, is disrupted during disease. To design successful therapies, it is critical to understand the complex brain cellular connections in AD and the temporal sequence of their disturbance. In this review, we discuss the interactions between different brain cells, from physiological conditions to their pathological reactions in AD, and how this basic knowledge can be crucial for developing new therapeutic strategies.

## 1 Introduction

Alzheimer’s disease (AD) is a devastating neurodegenerative disorder, expected to affect more than 100 million people worldwide by 2050 (Association, 2020), without sufficient nor efficient therapeutic strategies. Only in 2021, a disease-modifying drug, aducanumab, was approved by the Food and Drug Administration (FDA), although involved in controversy (Jeremic et al., 2021) due to its rapid approval and doubts about its effectiveness.

AD is characterized by the extracellular accumulation of amyloid-β peptide (Aβ) aggregates and filamentous intraneuronal inclusions of hyperphosphorylated tau protein (p-tau), which culminates in synaptic dysfunction and neuronal loss. Additional early pathological events in AD, preceding Aβ plaque deposition, include structural cerebrovascular alterations, as thickening of the basement membrane (BM) in brain microvessels, and early deficits in glucose uptake and in cerebral blood flow responses (Iturria-Medina et al., 2016). Furthermore, blood-brain barrier (BBB) dysfunction leads to the inability to remove neurotoxic substances from the central nervous system (CNS) and the infiltration of neurotoxic substances in the brain parenchyma, triggering neuroinflammation, which is associated with astrocyte and microglia activation, and results in the release of inflammatory mediators that cause neuronal damage (Solito and Sastre, 2012; Huang et al., 2020). These observations support that the regulation of the interaction between different brain cells is critical in AD (Henstridge et al., 2019), and it is therefore very important to increase the knowledge on this topic to fully uncover disease pathogenesis.

## 2 The multiple cells of the brain: Key intercellular interactions under physiological conditions

**FIGURE 1:**
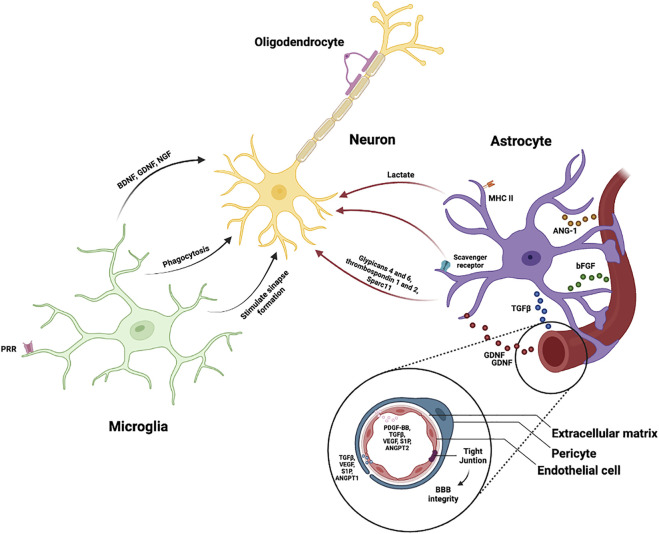
Cellular interactions in physiological conditions. All cell types in the brain interact in a complex network to ensure brain function. Microglia stimulate synapse formation, phagocyte neuronal corpses and synapses and generate neuroprotective factors (BDNF, GDNF and NGF). Astrocytes convert glucose in lactate which is then used to satisfy energy requirements of neurons, generate molecules that stimulate neuronal synapse development (Glypicans 4 and 6, thrombospondin 1 and 2, and Sparcl1) and prune synapses and engulf dying cells *via* scavenger receptors. Astrocytic endfeet participate in the regulation of angiogenesis and in the formation of endothelial cell-to-cell junctions by releasing soluble factors (GDNF, TGFβ, bFGF, ANG-1). Pericytes influence angiogenesis, extracellular matrix production, BBB function, wound healing, regulate immune cell infiltration and the blood flow, and the crosstalk between them and the endothelial cells is indispensable. Oligodendrocytes create myelin sheaths by wrapping myelin around axons.

A simple classification of the diverse cell populations in the CNS may be developed based on their distinct activity under normal and pathological situations. Distinct cell types in the nervous tissue develop many heterotypic interactions and engage in unique, frequently overlapping activities devoted to homeostasis maintenance (Nosi et al., 2021) (Figure 1).

### 2.1 Microglia

Microglia are resident macrophages that constitute around 10% of all cells in the CNS (Fakhoury, 2018). They are the initial line of cellular defense against invading infections and other forms of brain damage, because they are one of the first immune cells to become immunologically active during an inflammatory response (Solito and Sastre, 2012). Microglia remain in a resting state under normal conditions and are characterized morphologically by a small-shaped soma and highly ramified processes (Hristovska and Pascual, 2016). Resting state microglia allows continuous monitoring of the CNS for detection of pathogens and host-derived ligands, such as pathogen-associated molecular patterns (PAMPs) and danger-associated molecular patterns (DAMPs) (Solito and Sastre, 2012; Fakhoury, 2016). Pattern recognition receptors (PRRs) expressed on their molecular surface make them highly suitable for this function (Kigerl et al., 2014). Microglia get activated in response to invading pathogens and suffer morphological changes such as expansion of their soma and shortness of their cellular processes (Town et al., 2005). Activated microglia are critical in pathogen phagocytosis and in the elimination of cellular debris and degenerating cells at the lesion site (Sierra et al., 2014). Beyond their phagocytic activity, activated microglia assist in antigen presentation to T cells, orchestrating the communication between the innate and adaptive immune systems during an inflammatory response (Perry et al., 2010).

Several data point out that the microglia-mediated inflammatory response plays both harmful and helpful roles (Sierra et al., 2013; Kwon and Koh, 2020). Activated microglia generate inflammatory mediators such as cytokines, chemokines, inducible nitric oxide synthase (NOS), cyclooxygenase-2 (COX-2) and free radicals such as reactive oxygen species (ROS), which can disrupt neuronal processes and cause cellular disturbance (Solito and Sastre, 2012; Suzumura, 2013). When activated, microglia also generate a variety of neuroprotective factors that help in the prevention of neuronal damage, such as brain derived neurotrophic factor (BDNF), glial cell-derived neurotrophic factor (GDNF), and nerve growth factor (NGF) (Ding et al., 2004; Glezer et al., 2007; Suzumura, 2013). In summary, this inflammatory response can have a protective role, promoting tissue repair and eliminating cellular debris, however, this response can also produce a detrimental effect, as it will inhibit regeneration, leading to neurodegenerative diseases (Kwon and Koh, 2020).

This duality in microglia’s influence on immunological-mediated inflammation shows that these immune cells acquire different functional characteristics depending on their surrounding environment. Microglial cells have been generally categorized into proinflammatory M1 (neurotoxic) or anti-inflammatory M2 (neuroprotective) phenotypes based on their activation status (Tang and Le, 2016). The switch between these two phenotypes is triggered depending on the disease state, and is dependent on numerous factors such as insulin resistance, something that is interconnected with the progression of AD (Kwon and Koh, 2020). It should be emphasized, however, that this categorization is not uniformly supported by research findings, and there is still much to know about the mechanisms underlying microglia function throughout these different activation phases (Ransohoff, 2016). The M1 phenotype is classically triggered by Toll-like receptors (TLRs) or interferon-gamma (IFNγ), and it plays an important role in eliminating invading pathogens by releasing proinflammatory cytokines, which eventually cause neuronal damage in nearby tissues (Wang et al., 2015). The M2 phenotype, on the other hand, is alternatively triggered by IL-4 or IL-13 and is implicated in the production of large amounts of anti-inflammatory cytokines, therefore playing critical roles in tissue repair and angiogenesis (Wang et al., 2015). Interestingly, microglia that have transitioned to an M1 or M2 state may rapidly change their phenotype in order to adapt to their surrounding microenvironment, providing researchers the opportunity of addressing macrophage polarization imbalances for various therapeutic purposes (Wang et al., 2014). M1 macrophages, for example, can be polarized to M2-like macrophages by inhibiting the PI3K/AKT signaling pathway (Hyam et al., 2013) or the NF-κB, MAPK, and AKT pathways (Jang et al., 2013), while M2 macrophages can be converted into an M1 phenotype in response to lipopolysaccharide (LPS) and IFNγ (Mylonas et al., 2009).

Furthermore, microglia are a key component of the neurovascular unit (NVU) since they play an important role in angiogenesis and BBB function (Fantin et al., 2010). During fetal vascularization in the CNS, microglia support the stability and fusing of endothelial cells (ECs). Thus, activated microglia affect the integrity of the brain endothelial barrier directly and/or indirectly by increasing pro-inflammatory elements such as cytokines, chemokines and ROS.

### 2.2 Astrocytes

Astrocytes are the most prevalent glial subtype in the CNS and, such as microglia, play an important role in neuroinflammation modulation (Colombo and Farina, 2016). They have a star-shaped morphology with cellular processes extending from the soma and are also known as astroglia (Placone et al., 2015). Physiological activities of astrocytes in the healthy CNS include synaptic remodeling, neurotransmitter transmission, ion homeostasis, growth factor secretion, energy metabolism and oxidative stress control (Wyss-Coray and Rogers, 2012). Furthermore, astrocytes play an important role in the preservation and differentiation of dopaminergic neurons (Segura-Aguilar, 2015), and they have been linked to CNS diseases such as schizophrenia (McCullumsmith et al., 2016) and Parkinson’s disease (PD) (Gu et al., 2010), in which dopamine neurotransmission is implicated.

Due to their interaction with ECs and proximity to blood vessels, astrocytes contribute to the permeability of the BBB and also its maintenance (Abbott et al., 2006). Astrocyte endfeet are astrocytic terminal processes that cover the outer surface of the endothelium nearly entirely, creating a lacework of fine lamellae (Abbott, 2002). Astrocytic endfeet participate in the regulation of angiogenesis and also in the formation of endothelial cell-to-cell junctions by releasing soluble factors such as GDNF, angiopoetin-1 (ANG-1), basic fibroblast growth factor (bFGF), transforming growth factor-beta (TGFβ), and maintaining the function and structural integrity of the BBB (Cabezas et al., 2014). Several of the molecules mentioned above, as well as others such as IL-6 and hydrocortisone, have differentiating potential, implying that the BBB phenotype represents an enhanced state of differentiation that can be triggered and maintained by a variety of influences, some of which are derived from astrocytes (Abbott, 2002). Additionally, astrocytes play an important role in the induction of BBB features and functions by promoting the expression of enzymatic pathways, transporters (GLUT1, Pgp24, etc) and intermolecular junctions. (Lécuyer et al., 2016). Astrocytic endfeed also express aquaporin-4 and potassium channel, that control the ion and water balance at the BBB, allowing the support of its function (Michinaga and Koyama, 2019). Astrocytic expression of growth factors and cytokines also tightly regulates BBB permeability during inflammatory conditions, assisting in the regulation of immune cell passage into the CNS (Argaw et al., 2012).

When activated by pathogens, astrocytes release a diverse range of inflammatory cytokines that can be helpful or harmful. Furthermore, astrocytes produce major histocompatibility complex (MHC) class II molecules on their surface, acting as antigen-presenting cells for T cells (Gimsa et al., 2013). Astrocytes either repress (Gimsa et al., 2004) or promote (Saikali et al., 2010) T-cell functions depending on their surrounding environment and activation status. Although astrocytes are mostly neuroprotective (Bélanger and Magistretti, 2009), they contribute to the self-destructive environment by secreting chemokines and proinflammatory cytokines such as interleukin-1-alpha (IL-1α), interleukin-1-beta (IL-1β) and tumor necrosis factor-α (TNF-α) (Choi et al., 2014; Deng et al., 2014). Furthermore, astrocytes can physically interact with microglia, exerting substantial influence over their activation, phagocytic capability, and ability to produce inflammatory mediators such as TNF-α, IL-12, and inducible nitric oxide synthase (iNOS) (Fakhoury, 2018).

Recent research has linked astrocytes to glymphatic flux, a newly identified brain-wide network of perivascular spaces where cerebrospinal fluid (CSF) and interstitial solutes exchange (Wang et al., 2021). Astrocyte endfeet encircling the perivascular space form a physical barrier between these two compartments, and fluid and solutes that are not taken up by astrocytes move out of the perivascular space through the junctions in between astrocyte endfeet (Iliff et al., 2013a). This perivascular exchange, driven in part by vessel pulsation (Iliff et al., 2013b; Mestre et al., 2018b), is supported by the perivascular astroglial water channel aquaporin-4 (Iliff et al., 2012; Mestre et al., 2018a) and is faster in the sleeping brain than in the waking brain (Hablitz et al., 2019). Studies in rodents and humans suggest that glymphatic exchange dysfunction contributes to a variety of neurological conditions, including AD, assigning a cleaning role to astrocytes (Peng et al., 2016).

The brain has extremely high energy requirements, and glucose is the primary metabolic substrate for this organ (Magistretti and Allaman, 2013). Over the last two decades, an increasing body of data has supported the hypothesis that the majority of glucose taken up by the brain from the bloodstream is used by astrocytes, which convert it into lactate, which is then used to satisfy energy requirements of neurons (Pellerin and Magistretti, 1994, 2012). It has also been demonstrated that glutamate produced by activated neurons increases glycolysis in astrocytes, resulting in increased lactate generation (Pellerin and Magistretti, 1994). The close relationship between astrocytic metabolism and synaptic transmission indicates that astrocytes and neurons can function as a kind of syncytium, cross-regulating expression of proteins essential for energy metabolism and neurotransmitors production.

### 2.3 Oligodendrocytes

Oligodendrocytes are the myelinating cells of the CNS and promote neuronal transmission *via* saltatory action potential conductance across the Ranvier nodes. Oligodendrocytes create myelin sheaths by wrapping myelin around CNS axons (Kuhn et al., 2019). These myelin sheaths function as insulators for axons, allowing nerve impulses to travel quickly along them. Myelin sheaths limit ion leakage, allowing axons to maintain their electrical potential (Suminaite et al., 2019). As a result, a myelinated axon is far more efficient at signal transduction than an unmyelinated axon. A single oligodendrocyte has the ability to myelinate up to 50 axons (Kuhn et al., 2019).

Oligodendrocytes also provide buffer capacity for potassium, metabolic, trophic, and mechanic support to axons. They are considered particularly susceptible cells, and their number decreases dramatically (approximately 27%) in the aging brain (Pelvig et al., 2008). This cellular loss is represented in age-related myelin degradation, which may be observed in MRI scans of patients over the age of 50 (Bartzokis, 2011).

Overall, myelin content homeostasis in the CNS is based on the interplay between oligodendrocytes, astrocytes, and microglia. Myelin debris and undesirable myelin populations must be phagocytosed by microglia (Márquez-Ropero et al., 2020). However, neuroinflammation generated by any stress can impair remyelination and accelerate the loss of myelin-associated proteins (Nicol et al., 2015; Khurana et al., 2020; Kwon and Koh, 2020; Varas and Ortiz, 2020; Chavda et al., 2022). Therefore, remyelination is followed by the repair of myeline sheaths from axons, which protects them from disintegration.

Oligodendrocyte progenitor cells (OPCs) are a heterogeneous, multipotent population that arises during embryogenesis and remains as resident cells of the adult brain parenchyma. They are distinguished by the expression of the proteoglycan nerve-glial antigen NG2 and contribute up to 5% of mature brain cells. As main proliferating brain cells, OPCs have the ability to develop into oligodendrocytes, astrocytes, and potentially neurons, but this pluripotency is still being debated (Crawford et al., 2014).

### 2.4 Neurons

Neurons are the fundamental units of the brain that transport information throughout the human body. They help to coordinate all of the necessary functions of life by using electrical and chemical signals. Among other functions, neurons perform excitatory and inhibitory synaptic activity, promote electrical and chemical transmission, facilitate synaptic plasticity, and enable circuitry function. Neuronal activity requires a high level of energy, and a steady source of ATP is essential for the development, lifespan, and activity of neurons (Nicholls and Budd, 2000). The brain represents only 2% of total body weight, yet requires 20% of the body’s resting energy generation (Tomasi et al., 2013). In the brain, neurons use 70%–80% of the energy, while glial cells use the rest. Synapses are the principal locations of ATP utilization in the brain, where mitochondria supply 93% of the ATP requirement, with glycolysis providing the remaining 7% (Harris et al., 2012). Because neurons consume such high levels of energy, specific processes are required to keep energy homeostasis throughout the cell, specifically in distant synapses and axons (Sheng, 2014). This means that energy generation and consumption must be locally matched. As a result, mitochondria must be positioned near locations of high energy demand, such as pre- and post-synaptic domains, the axon initial segment, Ranvier nodes, and growth cones (Hollenbeck and Saxton, 2005). Axons and synapses are very malleable, and they remodel spontaneously and in response to activity, altering mitochondrial distribution. To adapt to different synaptic inputs, the location of mitochondria in neurons should be adjusted on quick time periods.

During development and neuronal circuit formation, synapse formation and remodeling is supported by both astrocytes and microglia. The majority of neuronal synapses are surrounded by and in contact with astrocytes. In studies over the last two decades, several molecules generated by astrocytes that stimulate neuronal synapse development have been found. Glypicans 4 and 6, thrombospondin 1 and 2, and hevin (Sparcl1) are among them (Allen and Eroglu, 2017). The molecules described so far have been demonstrated to stimulate both presynaptic assembly and postsynaptic maturation.

Synapse formation in adults and during cortical critical periods is stimulated by microglia, but the majority of data shows that their primary developmental activity is phagocytic. They engulf apoptotic neuronal corpses (Peri and Nüsslein-Volhard, 2008) and phagocytose synapses (Paolicelli et al., 2011). Important examples of their impact on synaptic functions include the loss of dendritic spines in the hippocampus, which is dependent on Trem2 signaling (Filipello et al., 2018). Surprisingly, astrocytes have been shown to prune synapses and engulf dying cells *via* scavenger receptors (Chung et al., 2013; Tasdemir-Yilmaz and Freeman, 2014). In the adult brain, astrocytes and microglia also support homeostatic neuronal function because both respond to and modulate neurotransmission (Vainchtein and Molofsky, 2020).

### 2.5 Endothelial cells

Endothelial cells are fundamental components of the vascular system and play an important role in the precise regulation of molecular transport across the BBB (Huang et al., 2020). The BBB is formed by a specialized EC layer, essential for its function and integrity. The BBB serves several important functions in the CNS. For instance, it regulates cerebral blood flow, which is necessary for CNS homeostasis. Second, it regulates the transport of glucose, oxygen, and other metabolites from the blood to the brain in order to keep neuronal circuits functioning properly. Third, the BBB enables the selective removal of metabolic waste from the brain through the brain vasculature (Daneman and Prat, 2015). Aside from these functions, the BBB serves as an endocrine target as well as an endocrine secretory tissue (Banks, 2019). Substances secreted by the neuroendocrine and endocrine systems influence the function of the BBB, which in turn influences brain function (Huang et al., 2020).

The BBB is responsible for maintaining the homeostatic balance in the brain by regulating the transport of substances. There are several types of transport across the BBB like: 1) passive diffusion: a spontaneous process that is dependent on a concentration gradient and where the molecules, like water-soluble nutrients and metabolites, diffuse passively across the endothelium into the brain (Kadry et al., 2020); 2) active efflux: it functions *via* efflux pumps, which are a group of ATP-binding cassette (ABC) proteins expressed on the blood-facing endothelial plasma membrane of the BBB and activated by ATP, as is the case of P-glycoprotein (Pgp), multidrug resistance-associated proteins (MRPs), and breast cancer resistance protein (BCRP) (Bellettato and Scarpa, 2018; Kadry et al., 2020); 3) carrier-mediated transport (CMT): is an energy-dependent mechanism in which specialized receptors on carrier membranes detect the target molecules such as amino acids, carbohydrates, monocarboxylic acids, fatty acids, hormones, nucleotides, organic anions, amines, choline, and vitamins and transport them across the cell (Mukherjee et al., 2017); 4) receptor-mediated transport: refers to the mechanism through which cells absorb substances by the inward budding of the plasma membrane, which is mediated by receptors on the cell’s surface as is the case of the Low-density lipoprotein Receptor-related Protein (LRP1) and the Receptor for Advanced Glycation End Products (RAGE) (Kadry et al., 2020). In pathological conditions, such as in AD, some transport mechanisms are described to be altered such as RAGE up-regulation and decreased expression of LRP1 and Pgp, leading to Aβ accumulation in the brain (Kadry et al., 2020).

Although capillaries with BBB properties vascularize most of the CNS, specific nuclei adjacent to the third and fourth ventricles, such as the subfornical organ, area postrema, pineal gland, and median eminence, contain vessels with much greater passive permeability (Ufnal and Skrzypecki, 2014). These circumventricular organs’ capillaries are continuous fenestrated vessels with high solute permeability, critical for the functions of these nuclei, which detect blood solute concentrations or secrete molecules into the blood (Daneman and Prat, 2015).

In contrast with ECs from other tissues, BBB ECs present particular features that allow them to effectively control the transport of ions, molecules, and cells between the blood and the brain (Daneman and Prat, 2015; Alajangi et al., 2022). Intercellular junctions between ECs play a critical role in vascular integrity and permeability barrier function (Vestweber, 2012). Tight junctions (TJs) and adherens junctions (AJs) are the two primary kinds of intercellular junctions found in ECs (Hirase and Node, 2012). TJs connect CNS ECs, limiting the paracellular flux of solutes (Greene and Campbell, 2016). When compared to peripheral ECs, CNS ECs have extremely low rates of transcytosis, which significantly limit vesicle-mediated transcellular solute movement (Profaci et al., 2020). This strong paracellular and transcellular barrier forms a polarized cell with distinct luminal and abluminal membrane compartments, allowing tight regulation of movement between the blood and the brain *via* regulated cellular transport features (Chow and Gu, 2015). The transporters expressed by CNS ECs fall into two types: efflux transporters and specific nutrient transporters (Alajangi et al., 2022). Efflux transporters transport a large variety of lipophilic molecules into the blood, while nutrient transporters guarantee the provision of particular nutrients across the BBB and help in waste elimination by transporting waste products from the CNS into the blood (Mittapalli et al., 2010). For the delivery of nutrients into the CNS parenchyma, CNS ECs express slc2a1GLUT1 for glucose transport, slc7a1 for cationic amino acid transport, slc16a1 and L-DOPA for lactate and pyruvate supply, and slc7a5 for neutral amino acid transport (Daneman and Prat, 2015; Alajangi et al., 2022). Several studies suggest that, at the BBB, ECs seem to rely on glycolysis to generate ATP (Kim et al., 2022), and to use mitochondrial respiration as secondary source of energy production. The reason for this is not clear, also because glycolysis is not efficient for ATP production, since only 2 ATP molecules per glucose molecule are generated, whereas mitochondrial respiration produces approximately 36 ATP molecules per glucose molecule. CNS ECs have more mitochondria than other ECs (Oldendorf et al., 1977), which is assumed to be important for generating ATP. EC switch to other metabolic processes, such as oxidative phosphorylation, may happen to ensure angiogenic activity and homeostasis of the endothelium and to drive the ion gradients required for transport activities, since this change carries a higher risk of ROS generation (Nag, 2011; Lee and Pienaar, 2014; Yetkin-Arik et al., 2019; Leung and Shi, 2022). CNS ECs also exhibit exceptionally low levels of leukocyte adhesion molecules (LAMs) as compared to ECs from other tissues, reducing the number of immune cells that enter the CNS (Daneman et al., 2010). Furthermore, variable vascular metabolism in CNS ECs is hypothesized to generate a barrier by changing the physical characteristics of molecules, which can influence their reactivity, solubility, and transport properties. The interplay of physical and molecular barrier properties, as well as particular transporters to provide needed nutrients, enables ECs to strictly regulate CNS homeostasis. A fundamental question is whether the BBB in different regions of the brain has particular characteristics that are essential for the local neuronal circuitry to function. Localized transport of certain nutrients, for example, might be critical for the growth or function of specific subclasses of neurons (Daneman and Prat, 2015).

Normal and adequate neuronal activity requires the preservation of a toxic-free brain microenvironment, and the BBB and its transporters play a critical role in this. As previously stated, ECs have unique properties that allow them to regulate the movement of molecules between the blood and the brain *via* the expression of transporters (Ramanathan et al., 2015). This regulation allows the maintenance of brain homeostasis, since the transporters will bind to waste molecules and compounds, eliminating them into the blood, for later elimination by other mechanisms. In this way, they are able to prevent any type of toxicity from being exerted by these compounds/molecules. At the BBB, ECs mainly present two distinct receptors that allow the transport of Aβ, LRP1 and RAGE (Alemi et al., 2016). RAGE is located on the luminal membrane of the endothelium and is involved in the entrance of Aβ into the brain (Deane et al., 2003). Differently, LRP1 is expressed on the abluminal side of brain vessels and is involved, for example, in the efflux of Aβ across the BBB, leading to its transport from the brain to blood and to its degradation in the kidneys and liver (Deane et al., 2009). Beyond LRP1, other receptors also appear to mediate the efflux of Aβ, such as low-density lipoprotein receptor-related protein 2 (LRP2) and Pgp (Jeynes and Provias, 2013). Under normal conditions Aβ levels in the brain maintain a homeostatic balance, but under certain conditions this balance is lost, leading to accumulation of this protein in the brain (Ramanathan et al., 2015).

Through the basal lamina, ECs make contact with astrocytic endfeet and pericytes, establishing the NVU with neurons (Hawkins and Davis, 2005; Stanimirovic and Friedman, 2012; Najjar et al., 2013). Among its functions in BBB maintenance, ECs are critical in bidirectional transport across the brain *via* ion transporters, protein and peptide carriers, and active efflux transport (Nag, 2011). Furthermore, the highly organized TJs and AJs present in the ECs limit the flow of polar substances such as hexose sugars, amino acids, nucleosides, monocarboxylic acids, and vitamins (Grammas et al., 2011; Mokgokong et al., 2014). To avoid the paracellular transport of numerous chemicals and ions, TJs integrity is required. Microbial infection, cancer, inflammatory reactions, stroke, AD, and PD are associated to its disruption (Stamatovic et al., 2008; Luissint et al., 2012). In conclusion, the cellular and molecular features of brain ECs are critical for maintaining BBB permeability *via* optimal ionic balance, junctional structure preservation, and proper interaction with NVU cells.

### 2.6 Pericytes

Pericytes are mural cells that sit on the abluminal surface of microvessels. Morphologically, they present an oval cell body with a great number of projections (Cabezas et al., 2014). In the NVU, pericytes are immersed in a thin layer of BM, which separates them from ECs, astrocyte endfeet and neurons (Wong et al., 2013). While most pericyte bodies and processes cannot connect to ECs due to the BM, interdigitations of pericyte and EC membranes can directly join in areas where the BM is absent, generating peg-and-socket connections. Furthermore, pericytes can connect with ECs *via* AJs and gap junctions, which are controlled by N-cadherin and connexin-43, respectively (Bonkowski et al., 2011; Winkler et al., 2011).

It has been shown that in developing and adult brains, pericytes influence angiogenesis, extracellular matrix production, BBB function, wound healing and regulate immune cell infiltration (Daneman and Prat, 2015). In addition, the capacity of pericytes to contract contributes to the regulation of blood flow by modulating capillary diameter (Winkler et al., 2011). Reports suggest that pericytes can also be multipotent stem cells of the CNS (Armulik et al., 2011). *In vitro*, they have shown the capacity to differentiate into chondrocytes, vascular smooth muscles cells, osteoblasts and skeletal muscle, showing that pericytes have a promising therapeutic role in CNS injuries and other pathologies (Armulik et al., 2010; Lange et al., 2013; Cabezas et al., 2014).

Pericytes in the CNS have been found to exhibit distinct features when compared to pericytes in other tissues. CNS pericytes are derived from the neural crest, in contrast with pericytes in many peripheral tissues, which are derived from the mesoderm (Majesky, 2007). One of the key questions in pericyte biology is whether there are distinct subgroups of pericytes with distinct roles. Because there are no identifying markers, it is unknown whether all of the numerous tasks assigned to pericytes are accomplished by the same cells, different subsets of pericytes, or even non-pericyte cells that reside close to the vasculature. Recent data obtained by Single-cell RNA (scRNA) sequencing of the adult mouse brain relative to vascular and vessel-associated cell types defined 15 cell clusters, including pericytes, three types of vascular smooth muscle cells, microglia, two types of fibroblast-like cells, oligodendrocyte-lineage cells, six types of endothelial cells and astrocytes (He et al., 2018). In this work, authors also report a strong variation in gene expression levels between individual cells, including pericytes, concluding that the variation of gene expression between cells of the same population is a stochastic event. The existence or nonexistence of lysosome granules in the cytoplasm has often been used to characterize pericytes. In the brain, two main types of pericytes are present, granular (95 percent of total pericytes) and agranular. Changes in granular pericytes have been linked to amyloid deposition and lipid accumulation in human brain cultures, indicating the relevance of pericyte changes in AD and other diseases (Castejón, 2011; Cabezas et al., 2014).

The interactions between astrocytes and pericytes are essential and it has been shown that both of them are fundamental for brain vasculogenesis and BBB maintenance probably through the activation of PDGFRB signaling (Bonkowski et al., 2011). Furthermore, both pericytes and astrocytes play a role in the maintenance of EC TJs by regulating proteins such as occludin, claudin, and zona occludens-1 (ZO-1) (Bonkowski et al., 2011). This finding indicates that astrocyte-pericyte communication is important in brain function. However, more research is needed to fully comprehend the implications of the aforementioned interactions throughout neurodegenerative disorders.

## 3 Disruption of key brain intercellular interactions—Contribution to AD pathogenesis

**FIGURE 2:**
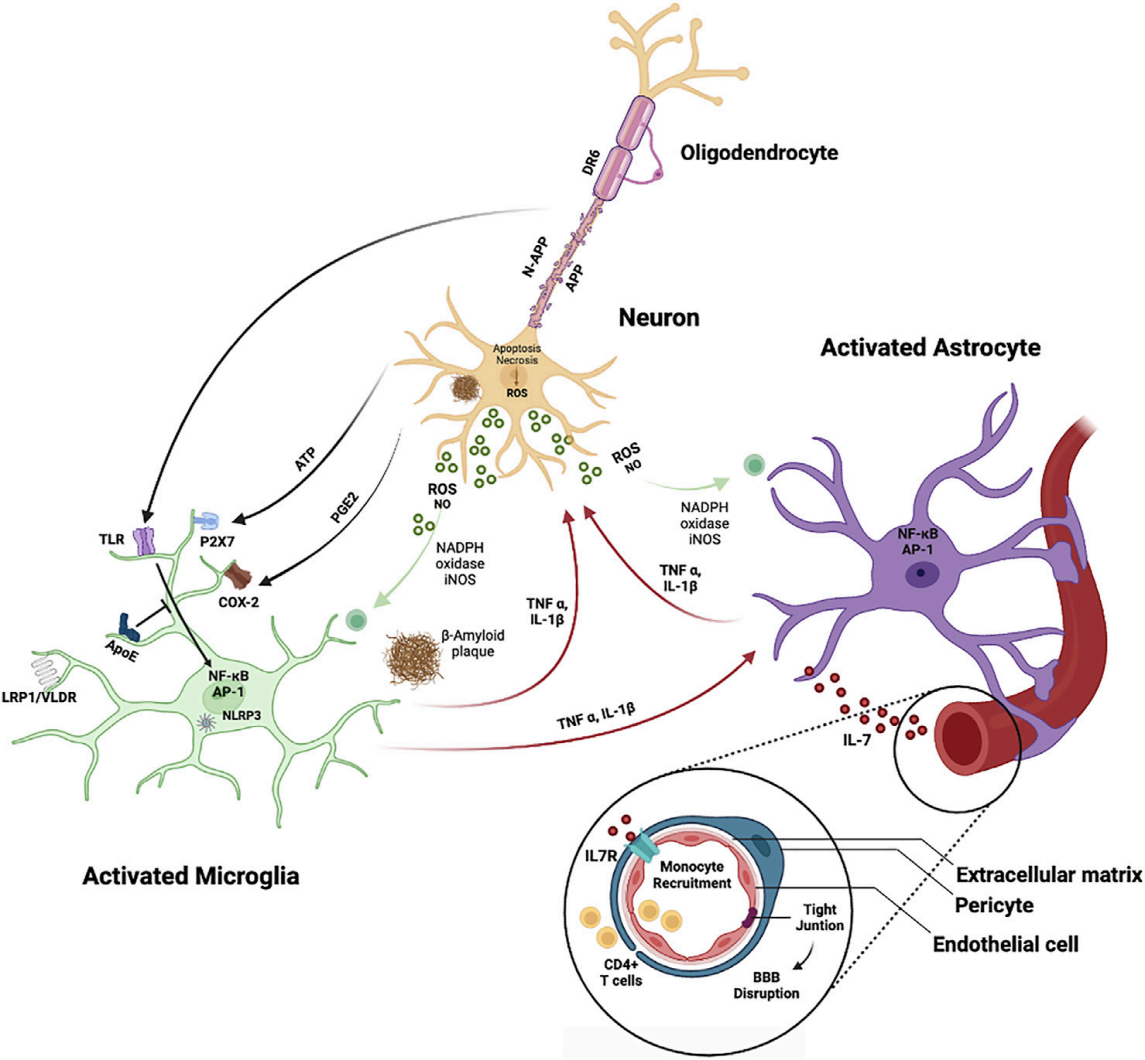
Cellular interactions in Alzheimer’s disease (AD). Cleavage of the amyloid precursor protein (APP) results in the formation of amyloid-β peptide (Aβ) that aggregates and accumulates forming the amyloid plaques. These aggregates activate microglia cells *via* Toll-like receptors (TLRs) and receptor for advanced glycation end products (RAGE), altering the transcriptional program of the microglia cells and stimulating the expression of transcription factors such as AP-1 and NF-κB, which in turn trigger the production of reactive oxygen species (ROS) and inflammatory cytokines. These cytokines enhance the proinflammatory environment by stimulating astrocytes, which act directly on the neuronal population, causing neuronal cell death. Neuronal apoptosis results in release of ATP, which then activates microglia *via* the purinergic P2X7 receptor, initiating an autostimulatory loop that induces T-cell infiltration. In AD, endothelial cells and pericytes are dysregulated, causing BBB disruption, and the number of oligodendrocytes is reduced, resulting in diminished neuron myelination.

As described so far, the brain is constituted by multiple cell types that work together and rely on each other to maintain a healthy brain. It is reasonable to think that these cells react to early changes that occur in the AD brain, to protect from damage. However, when balance cannot be restored, the same cells may impose damaging effects (Figure 2).

### 3.1 Microglia triggers neuroinflammation

A large number of studies suggest that inflammatory mechanisms are modified in AD due to an increased immune response (Wyss-Coray and Rogers, 2012; Meraz-Ríos et al., 2013). The fact that inflammatory pathways may accelerate neuronal loss and cognitive decline (Holmes et al., 2003; Cunningham et al., 2005), as well as data linking polymorphic differences in inflammatory cytokines to AD (Nicoll et al., 2000; McCusker et al., 2001; Hayes et al., 2004), support the involvement of microglia in AD pathogenesis. During an inflammatory response, microglia are one of the first immune cells to be activated and attracted to the injury site. Understanding their role in AD might not only help to understand the cellular and molecular mechanisms behind neurodegeneration, but can also allow new treatment possibilities. The earliest investigations linking microglia to AD found that these immune cells are heavily involved in the production of Aβ plaques in the brains of AD patients (Wyss-Coray and Rogers, 2012; Meraz-Ríos et al., 2013). Data from animal models of AD have recently revealed the existence of activated microglia near regions of Aβ deposition, indicating that these glial cells may directly interact with Aβ and modulate its levels in the brain (Holmes et al., 2003; Cunningham et al., 2005). Genetic investigations demonstrate that a null mutation in the Triggering Receptor Expressed on Myeloid Cells 2 (TREM2) gene, which is primarily expressed by microglia in the CNS, is related with a diminished capacity of microglia to enclose amyloid deposits (Yuan et al., 2016). Furthermore, genetic deletion of the complement factors C1q and C3, as well as the microglial complement receptor CR3, decrease the number of phagocytic microglia and the amount of early synapse loss, implying that complement activation may be an early modulator of plaque-associated synapse loss in AD by activating phagocytic microglia (Hong et al., 2016). Microglia and CR3 are also important in Aβ homeostasis, since CR3 ablation in APP-transgenic mice results in lower Aβ accumulation, probably due to enhanced production of Aβ-degrading enzymes and improved capacity of microglia to degrade extracellular Aβ (Czirr et al., 2017). Despite tremendous advances in understanding the relationship between microglia and Aβ in AD, whether Aβ accumulation in the brain precedes microglia activation still remains a source of discussion (Jung et al., 2015; Fakhoury, 2018).

Microglia interact with Aβ, but also with APP, *via* specific PRRs highly expressed on their surface, such as CD14, CD36, and TLRs (Jana et al., 2008; Richard et al., 2008; Reed-Geaghan et al., 2009). This interaction is necessary for the phenotypic activation of microglia and the induction of phagocytosis, which culminates in Aβ clearance from the brain (Lee and Landreth, 2010; Solito and Sastre, 2012). Microglia can phagocytize Aβ aggregates *via* an ensemble of cell surface receptors, including class A scavenger receptor (SR-A) (Khoury et al., 1996; Paresce et al., 1996), class B scavenger receptor (CD36), CD14, CD47, TLR2, TLR4, TLR6, and TLR9 (Bamberger et al., 2003; Reed-Geaghan et al., 2009; Stewart et al., 2010). Inflammatory stimuli, such as LPS, also activate microglia, promoting Aβ degradation (Qiu et al., 1997). Microglia dysfunction in transgenic mice enhances the progression of AD and leads to greater Aβ accumulation in the brain, which is consistent with the idea that microglia are involved in Aβ clearance (El Khoury et al., 2007; Krabbe et al., 2013). Apart from being advantageous to the host, activation of microglia by Aβ or APP results in an up-regulation of inflammatory mediators such as iNOS, tumor necrosis factor-alpha (TNF-γ), IL-1β and IL-6, eventually leading to an increased inflammatory response and significant neuronal death (Cai et al., 2016; Sajja et al., 2016). Taken together, the findings show that microglia perform multiple tasks in AD in a context-dependent way. While mild activation of microglia provides protection by allowing Aβ clearance in the brain, over activation of these cells by Aβ or APP may result in an excessive inflammatory response, aggravating the neurodegenerative processes in AD.

Regardless of substantial advancements in research, only a few studies have looked into the association between microglia and the accumulation of NFTs in AD. One of them suggests that a null mutation in the TREM2 gene is also related to severe neuritic tau hyperphosphorylation (Yuan et al., 2016). Although some studies appear to suggest that microglia play a role in the internalization and degradation of tau, the main component of NFTs (Bolós et al., 2015; Luo et al., 2015), others suggest that pro-inflammatory cytokines, like IL-1 and IL-6, produced by active microglia cause tau phosphorylation, which promotes the development of NFTs (Merighi et al., 2022). In this sense, more research is needed to get a better understanding of the molecular processes behind microglia’s participation in NFTs accumulation.

Activation of microglia when followed by uncontrolled neuroinflammation is often associated with a disruption of the BBB (Lassman et al., 2012). A recent study found that during inflammation, vessel-associated microglia safeguard BBB integrity by expressing the tight-junction protein Claudin-5 (Haruwaka et al., 2019). Microglia, on the other hand, exhibit a more active phenotype with chronic inflammation, resulting in impaired BBB function (Haruwaka et al., 2019). When microglia are activated around Aβ deposits and release inflammatory cytokines such as IL-1α, IL-1β, IL-6, TNF-α and TNF-β, BBB integrity and permeability can be compromised, resulting in increased neutrophil migration through the BBB into the brain (Allen et al., 2012; Zenaro et al., 2015). The activation of microglial cells also results in the formation of reactive oxygen and nitrogen species, which causes neurotoxicity and BBB disruption (Block, 2008; Sumi et al., 2010). Glial-mediated inflammation performs both detrimental and beneficial functions in AD. It is still unclear whether the glial-mediated inflammatory response observed in AD is a result or a cause of neurodegeneration. A deeper knowledge of the role of microglia in the regulation of AD pathogenesis is required in order to develop new treatment options.

### 3.2 Astrocyte-microglia crosstalk in inflammation

In the AD brain, astrocytes face a variety of morphological alterations, as well as changes in their expression profiles, and are triggered into a reactive astrocyte state, which is one of the key hallmarks of AD pathology (Cai et al., 2017). Astrocytes perform antagonistic roles in the pathogenesis of AD. On one hand, they can play the typical neuroprotective role, resulting in Aβ clearance and the creation of a barrier around plaques. On the other hand, astrocytes can have a neurotoxic impact. There is a reciprocal modulation between microglia and astrocytes, which ultimately results in the change of phenotypes of both cell types to a more neurotoxic phenotype (Dionisio-Santos et al., 2019). Cross-talk between astrocytes and microglia causes a persistent inflammatory response as well as the production of gliotransmitters, both of which contribute to neurodegeneration (Yi et al., 2016; Perez-Nievas and Serrano-Pozo, 2018). The existence of this bidirectional communication between microglia and astrocytes plays a very important role in the modulation of inflammation in the CNS, and it is something that is compromised during a neurodegenerative disease in which neuroinflammation only tends to worsen (Linnerbauer et al., 2020). Reactive astrocytes in AD also have decreased glutamate transporter 1 (GLT1) expression and/or mislocalization, which impairs glutamate reuptake at synapses and causes neuronal injury (Hefendehl et al., 2016). Furthermore, GLT1 deficiency has been associated to the deterioration of the cognitive function in AD patients (Masliah et al., 1996).

During AD pathogenesis, astrocytes are engaged in the generation and clearance of Aβ peptides (Liu et al., 2017), however, the mechanisms by which astrocytes react with Aβ are largely unknown. Astrocytes express a diverse set of receptors that recognize and bind to Aβ, including RAGE, lipoprotein receptor-related proteins (LRPs), membrane-associated proteoglycans, and scavenger receptor-like receptors (Ries and Sastre, 2016). Internalization of Aβ occurs *via* the RAGE and LRP1 pathways (Liu et al., 2016); however, astrocytes also secrete different extracellular Aβ degrading enzymes, including neprilysin (Yamamoto et al., 2014, 2016, 2017), insulin-degrading enzyme (Son et al., 2015, 2016), matrix metalloproteinase (MMP)-2, and MMP-9 (Yin et al., 2006). Apoliprotein E (ApoE), a protein required for Aβ clearance across the BBB, is also mostly produced and released by astrocytes (Zhang et al., 2016; Suidan and Ramaswamy, 2019), but some studies indicate that microglia and neurons are also able to produce this protein (Huang et al., 2004; Zhang et al., 2016). ApoE is involved in several processes, including lipid transport to neurons (Nathan et al., 1994; Pitas et al., 1998), synaptogenesis (Levi et al., 2003), cerebrovascular integrity and cerebral blood flow (Bell et al., 2012; Koizumi et al., 2018), hippocampal neurogenesis (Tensaouti et al., 2018), neuroimmune modulation (Zhu et al., 2012), redistribution of cholesterol and phospholipids within the brain (Lee and Landreth, 2010) and amyloid clearance (Riddell et al., 2007; Cramer et al., 2012). The three common human isoforms, apoE2, apoE3 and apoE4, have a significant genotype-dependent influence on apoE function, including lipid transport and amyloid clearance *via* the BBB (Liu et al., 2013; Suidan and Ramaswamy, 2019). Importantly, these three prevalent human apoE isoforms have a substantial genotype influence on the risk and age of onset for sporadic and late-onset AD. Carriers of an ε4 allele have an increased risk of developing AD, while those with an ε2 allele are protected (Suidan and Ramaswamy, 2019). Several studies have shown that microglial cells express high levels of the ATP-binding cassette transporter subfamily A member (ABCA1), which is an efflux pump for cholesterol and phospholipids that contribute to ApoE lipidation in the brain (Fitz et al., 2012; Lupton et al., 2013). The rate of ApoE lipidation is tightly involved in mediating Aβ uptake, thus contributing to Aβ clearance through the BBB *via* endothelial LRP1 (Thériault et al., 2015).

After Aβ deposition, aberrant astrocytic activity disrupts Aβ internalization and secretion of extracellular Aβ degrading enzymes, resulting in abnormalities in Aβ clearance and in the formation of amyloid plaques and NFTs in AD brains (Olabarria et al., 2010). For example, poor RAGE/LRP1-dependent Aβ clearance by astrocytes causes a disparity between Aβ generation and clearance (Ueno et al., 2014; Liu et al., 2016). The number of active astrocytes in the parahippocampal cortex of AD patients correlates with the number of tangles and the stage of NFT development, indicating that astrocyte activation plays a role in the development of NFTs in AD (SHENG et al., 1997). Additionally, thrombin, a serine protease produced by astrocytes and microglia, accumulates in NFTs (Arai et al., 2006) and is involved in tau cleavage (Olesen, 1994). Although these findings suggest a possible mechanistic pathway through which activated astrocytes may reduce the neurodegenerative processes in AD, additional research is needed to better comprehend the molecular mechanisms behind the formation and evolution of NFTs. Aβ also activates astroglial nuclear factor-kappa B (NF-κB) and complement signaling, impairing synaptic density and dendritic morphology (Lian et al., 2015), and increases astrocyte generation of inflammatory mediators such as, IL-1α, IL-1β, IL-6 and TNF-α, in response to scavenger receptor ligands (Murgas et al., 2012) and LPS (Forloni et al., 1997), contributing to the neurodegenerative changes seen in AD (Sajja et al., 2016).

Furthermore, astrocyte endfeet depolarization in AD brains may lead to the degradation of BBB integrity (Yang et al., 2011). The structural abnormalities in astrocytes around Aβ deposits in both the brain parenchyma and cerebral blood vessels have been documented in studies on cortex biopsies from AD brains (Wisniewski et al., 1989). In AD patients and murine AD models linked with cerebral amyloid angiopathy (CAA), astrocyte endfeet that surround vascular Aβ deposits experience structural alterations including swelling, retraction, and separation.

During AD, astrocytes develop multiple states that are linked with either gain or loss of function, contributing to neuroinflammation and neurodegeneration. A comprehensive description of these cellular states, will allow understanding how astrocytes evolve throughout the disease, and in the near future, we may be able to link different astroglial states to specific stages of AD, potentially leading to novel biomarkers and targets for therapeutic intervention.

### 3.3 Oligodendrocyte dysregulation and axon demyelination

Demyelination is common in AD, and has been designed as a predictor for AD onset and subsequent neurodegeneration (Nasrabady et al., 2018). The loss of myelin integrity in the hippocampus seen in 3xTg-AD mouse models suggests that oligodendrocytes are assaulted pathophysiologically during disease progression (Desai et al., 2010). The protein monocarboxylic acid transporter 1 (MCT1) assists oligodendrocytes in sustaining metabolic support to neurons, and AD models revealed a substantial decrease in brain MCT1 levels, indicating axon injury and neuron loss (Mot et al., 2018). MRI and advanced positron emission tomography revealed increasing white matter damage in the frontal and temporal lobes of the brain during the early, preclinical, and later phases of AD, before any obvious clinical indications (Khan, 2018). Furthermore, in AD, there is a common impairment in the recruitment, migration, proliferation, differentiation, and regeneration of chondroitin sulfate proteoglycan Neural/glial antigen 2 (NG2)-expressing OPCs into oligodendrocytes. The increased OPC proliferation has a beneficial effect, in which the NG2 cells that surround Aβ plaques internalize and degrade Aβ (Li et al., 2013). NG2 cells may also be seen nearby microglia and astrocytes, showing a tight relationship with the neuroinflammation triggered by AD (Nirzhor et al., 2018).

LPS, which plays an important regulatory function in myelination, also co-localizes with amyloid plaques and perivascular amyloid in the AD brain (Zhan et al., 2018). The disruption of the BBB in AD patients, enables the passage of LPS into the brain and its interaction with the TLR4/CD14 receptors on the peripheral monocytes/macrophages, neutrophils, and brain microglia. Following NF-kB activation, cytokines such as IL-1α, IL-1β, IL-6, and TNF-α are produced, which damage the axon and myelin sheath and potentiate AD pathogenesis (Zhan et al., 2018). Previous studies have shown that aberrant myelination precedes axon abnormalities and axon transport before the emergence of Aβ and tau pathology (Cai and Xiao, 2016). LPS binds directly to oligodendrocytes in both the white and gray matter, increasing cytokine and free radical production. Furthermore, extremely high cytokine levels cause OPC mortality and exacerbate mature oligodendrocyte and myelin sheath destruction, resulting in abnormal myelin aggregation in AD (Schmitz and Chew, 2008).

The formation of oligodendrocytes is tightly linked to neuregulin axonal expression, which is strictly controlled by caspase-6 activation. AD-induced myelin morphology changes are connected to caspase-6-dependent neuregulin type III cleavage (Hu et al., 2016). The overexpression of neuregulin III causes hypermyelination, affects myelin morphology, changes electrical impulses along the axon and at nerve terminals, and ultimately causes a spatio-temporal cognitive deficit development (Hu et al., 2016).

Injured oligodendrocytes and myelin sheath, deteriorated axons, and aggregated Aβ, all contribute to the formation and deposition of Aβ plaques (Papuć and Rejdak, 2020). Incubation of cultured oligodendrocytes with Aβ results in apoptotic cell death (Xu et al., 2001), and injection of Aβ into the rat corpus callosum results in axon damage, oligodendrocyte death, and reactive gliosis around the lesion (Jantaratnotai et al., 2003). Aβ activates sphingomyelinase (NSMase)-ceramide in the cell membrane *via* an oxidative process, producing ceramide with pro-apoptotic characteristics and causing oligodendrocyte malfunction (Jana et al., 2009).

Inflammatory circumstances, such as those seen in AD, can hinder the remyelination process and prevent OPCs from developing into oligodendrocytes (Miron et al., 2013). Furthermore, inflammation and oxidative stress have been identified as the two primary processes linking oligodendrocyte death to NFTs (Cai and Xiao, 2016). Although not fully confirmed, these hypothesized oligodendrocyte activities contribute to highlight the important role of interactions between different cell types in disease.

### 3.4 Endothelial cell dysfunction and BBB disruption

Post-mortem analysis showed that TJ proteins, occludin, claudin-5, and ZO-1 were significantly decreased in human brain capillaries with CAA, which was associated with increased fibrinogen leakages in the brain parenchyma (Carrano et al., 2011, 2012). Furthermore, changes in cerebral TJs were reported in the AD model 5XFAD mice. TJ lengths in these animals were considerably shorter than those in littermate control mice, according to electron microscopy (Kook et al., 2012). Consistent with those findings, exposure to Aβ_42_, particularly the oligomeric form, dramatically reduced levels of occludin, claudin-5, and ZO-1 (Kook et al., 2012; Wan et al., 2015) and disrupted barrier integrity (Wan et al., 2015), in the cellular model of mouse brain ECs, the bEnd.3 cell line. Other studies have shown that incubation with Aβ_40_ and Aβ_42_ reduces occludin in human brain endothelial hCMEC/D3 cells (Tai et al., 2010) and in primary rat brain ECs (Marco and Skaper, 2006), respectively. Furthermore, in a cystathionine-*β*-synthase heterozygote mouse model (Cbs+/−, a genetic model of hyperhomocysteinemia), hyperhomocysteinemia has been reported to cause a considerable reduction in VE-cadherin in the cerebrovasculature, as well as increased BBB permeability and cerebrovascular deposition of Aβ and fibrinogen (Muradashvili et al., 2014). As a result, Aβ is likely to affect the structure of TJs and AJs in ECs, impairing their barrier function.

While glucose transporter 1 (GLUT1) is a type 3 integral transmembrane protein that is specifically expressed in brain ECs, it is drastically diminished in the brain microvessels of AD patients and amyloid mice models (Hooijmans et al., 2007; Merlini et al., 2011). Endothelial GLUT1 loss causes early BBB disruption in mice, as shown by a decrease in TJ proteins and extravascular accumulation of fibrinogen and IgG (Winkler et al., 2015). Furthermore, in an amyloid mouse model that overexpresses APP, GLUT1 deficiency causes cerebral microvascular degeneration followed by accelerated Aβ pathology (Winkler et al., 2015). Thus, a decrease in GLUT1 in microvessels during AD might contribute to disease pathogenesis. This deficiency in GLUT1 at the BBB is also associated with the development of type 3 diabetes (T3DM) in AD patients, establishing a link between insulin resistance in the brain and AD. In T3DM there is a progressive insulin resistance in the brain, which ends up having neurocognitive consequences, since there is an impairment of the insulin signaling centers, an accumulation of neurotoxins and neuronal stress, which will lead to neurodegeneration (Nguyen et al., 2020). Given this, diabetes affects the metabolism and cognitive functions of the brain, having an influence on memory processing and synaptic communication, characteristics that are also found in AD (Leszek et al., 2017; Nguyen et al., 2020). Typical features of AD, such as neuroinflammation, the formation of toxic Aβ aggregates, and the hyperphosphorylation of the Tau protein, are related to central insulin resistance, which leads to the conclusion that in fact there is an interconnection between the triggering factors of both diseases (Nguyen et al., 2020).

In 1993 Torre and Mussivand postulated the vascular hypothesis as an explanation for the pathophysiology of AD (De la Torre and Mussivand, 1993). This theory proposes that blood vessels are the starting point for a number of pathogenic pathways that lead to neuronal damage and dementia. Destruction of the BBB organization, decreased cerebral blood flow and glucose metabolism, and the establishment of an inflammatory context would thus be responsible for any subsequent neuronal damage, since these factors promote Aβ aggregation in the brain. The discovery of a link between neurodegeneration and vascular dysfunction pathways has resulted in new drug targets and therapeutic approaches for AD (Rius-Pérez et al., 2018).

In addition to sustaining endothelial barrier formation, the BBB acts as an interface between the peripheral immune system and the CNS immune system (Prinz and Priller, 2017). When cerebrovascular ECs and circulating leucocytes are activated as part of immune responses, the expression of adhesion molecules and chemoattractant productions are up-regulated in those cells, allowing circulating immune cells to cross the BBB into brain parenchyma (Grammas, 2011; Zenaro et al., 2017). Through the synthesis and release of proinflammatory cytokines, ROS, and active proteases, infiltrating immune cells are likely to cause structural changes in the BBB. Reciprocal stimulation of cells in the NVU, namely glial cells, and their release of cytotoxic mediators may similarly impact the BBB, prolonging endothelial inflammation. While leukocytes such lymphocytes, monocytes, and neutrophils are expected to cross the BBB and enter the brain in AD (Rossi et al., 2011), neutrophil reduction has been found to enhance cognitive performance and diminish AD-related pathology in amyloid model mice (Zenaro et al., 2015). Thus, modulating vascular inflammation and/or leukocyte trafficking across the BBB may have therapeutic potential in AD.

Under physiological or pathophysiological conditions, autoantibodies are produced. Physiologically, humans produce natural autoantibodies (autoantigens) that recognize self-antigens to assist in the identification and clearance of dead and dying cells (Elkon and Silverman, 2012). Natural autoantibodies promote phagocytosis of apoptotic cells while suppressing inflammatory pathways. As a result, natural autoantibodies play an important role in reducing inflammation and maintaining immune tolerance (Elkon and Silverman, 2012). Immune tolerance breakdown is a major mechanism that leads to pathogenic autoantibody production and autoimmune diseases. Pathogenic autoantibodies initiate and maintain the inflammatory cascade that causes tissue damage by binding to self-antigens with high affinity. There are over 80 different types of autoimmune diseases (Cho and Feldman, 2015), and several lines of evidence suggest that AD may also be an autoimmune disease (D’Andrea, 2005). Numerous studies have found autoantibodies against various molecules in AD patients, including autoantibodies against Aβ, tau, glial markers, vasculature-related molecules, cellular enzymes, and neurotransmitters and related receptors (Wu and Li, 2016).

Autoantibodies can be both harmful and protective. Autoantibodies interfere with cellular function and cause a severe inflammatory response, resulting in brain tissue damage and autoimmune disease in pathological conditions. Autoantibodies confer immune tolerance, reduce inflammation, and assist in the clearance of toxic proteins under physiological conditions. Autoantibodies’ beneficial effects have served as the foundation for the development of anti-Aβ and anti-tau immunotherapies. However, many autoantibodies’ precise roles are currently unknown. Some autoantibodies are specifically associated with disease status and thus may serve as diagnostic/prognostic biomarkers for AD. In particular, new “autoantibomic” techniques using protein or peptide/peptide arrays show great promise for the diagnosis of AD (Wu and Li, 2016).

### 3.5 Pericyte impact on the vascular component

Independent studies have found pericyte loss and/or degeneration in the hippocampus and cortex of AD patients (Baloyannis and Baloyannis, 2012; Sengillo et al., 2013). Pericytes have a high number of intracellular inclusions, pinocytotic vesicles, large lipid granules, and mitochondrial abnormalities, implying cellular malfunction and/or degeneration (Farkas and Luiten, 2001; Baloyannis and Baloyannis, 2012). Capillary reductions, as well as general dilatation and tortuosity of surviving vessels, are related with pericyte degeneration (Baloyannis and Baloyannis, 2012). Although these findings are restricted to postmortem tissue, pericyte dysfunction and/or loss are linked to two critical features of AD vascular pathology: vascular regression and altered vascular permeability.

Recent studies suggest that the loss and/or malfunction of brain pericytes may exacerbate the *in vivo* AD-like neurodegenerative pathway. In mouse models, the loss of brain pericytes is enough to cause neurodegenerative alterations in the absence of Aβ *via* two key pathways: BBB disruption and hypoperfusion (Bell et al., 2010; Winkler et al., 2011, 2012). On one hand, a loss of pericytes increases vascular permeability *via* a damaged BBB. As a result, various blood-derived neurotoxic and vasculotoxic compounds, including fibrin (Paul et al., 2007), thrombin (Tripathy et al., 2013), plasmin (Chen and Strickland, 1997), hemoglobin-derived iron and ROS, accumulate in AD brain (Wu et al., 2002; Hua et al., 2007). On the other hand, loss of pericytes causes regression of brain microvessels, especially capillaries. This causes prolonged perfusion stress and hypoxia. Toxin accumulation and hypoxia could have an impact at the neuronal interface, resulting in damage, malfunction, and, eventually, cell death (Bell et al., 2010).

Loss of brain pericytes also leads to Aβ-dependent toxicity that contributes to the neurodegeneration seen in AD. A recent study used pericyte deficient mutants (Pdgfrβ^+/−^ mice) crossed with mice that overexpress the Swedish mutation of human APP (APP^sw/0^) (Sagare et al., 2013). While APP^sw/0^ mice accumulated parenchymal and vascular Aβ leading to amyloid plaques and memory deficits, but did not develop tau pathology and/or neuronal loss (Hsiao et al., 1996; Spires and Hyman, 2005), APP^sw/0^; Pdgfrβ^+/-^ animals displayed increased deposition of soluble Aβ_40_ and Aβ_42_ species due to impaired pericyte-dependent clearance of soluble Aβ species from the brain interstitial fluid. Moreover, in the double transgenic mice, impaired soluble Aβ clearance subsequently led to rapid deposition of insoluble Aβ, culminating in CAA and parenchymal β-amyloid plaques. At the age of 9 months, APP^sw/0^;Pdgfrβ^+/-^ animals exhibited tau pathology, which included neuronal accumulation of hyperphosphorylated tau species, caspase-cleaved tau, and tau aggregates (Sagare et al., 2013). Importantly, tau pathology was not found in either APP^sw/0^ or Pdgfrβ^+/-^ mice at this relatively early disease stage, suggesting that both pericyte-driven vascular damage and Aβ elevations must be present to trigger early tau pathology. In terms of neuronal loss, Pdgfrβ^+/-^ animals demonstrated more moderate neuronal loss as a result of direct vascular damage, as previously documented (Bell et al., 2010). The amount of neuronal dysfunction and/or loss, as well as behavioral impairment on hippocampal-dependent activities, was significantly greater in animals with an enhanced pericyte loss (Sagare et al., 2013). This study suggests that pericytes influence multiple steps of the pathogenic cascade of AD-like neurodegeneration and may be considered as new therapeutic targets to modify the progression of AD.

### 3.6 Neuronal loss and dysfunction

Neurons express a huge number of molecules that defend them from inflammatory attacks and against the induction of neurological disorders. But, in AD, neurons have been found to be damaged and dysfunctional.

Cerebral perfusion is impaired in AD due to poor brain interstitial fluid drainage caused by perivascular space loss and Aβ accumulation. Aβ has the ability to bind to specific neuronal receptors and induce toxicity, which is mostly caused by oxidative stress. Reduced cerebral blood flow can cause neuronal dysfunction by changing proteins involved in synaptic plasticity (Iadecola, 2004) and favoring anaerobic brain metabolism. Reduced cerebral blood flow can also reduce ATP production, which is essential to sustain the Na^+^, K^+^-ATPase pump and the action potentials required for proper neuronal excitability (Iadecola, 2013). Transient oxygen and glucose deprivation have been demonstrated to produce neuronal excitotoxicity and subsequent neuronal death (Shabir et al., 2018). Oxygen deprivation can affect pH and water-electrolyte balance, resulting in edema, white matter lesions, and increased glutamate and Aβ levels in the brain (Zlokovic, 2011). A decrease in the levels of glucose transporters in both neurons (GLUT1) and BBB (GLUT3) in AD patients, raised the hypothesis that these transporters may have a role in the impairment of glucose uptake and metabolism in the brain, which can trigger the phosphorylation of tau protein (Michalicova et al., 2017). The tau protein is a microtubule associated protein and is involved in mitochondrial axonal transport in neurons. The regulation of tau phosphorylation influences its ability to bind microtubules (Wyss-Coray and Rogers, 2012). During AD progression, hyperphosphorylated tau, dissociates from microtubules, and accumulates intracellularly as NFTs (Grundke-Iqbal et al., 1986; Nukina and Ihara, 1986; Mondragón-Rodríguez et al., 2008, 2010). This impairs mitochondrial axonal transport between the cell body and the synapses, resulting in energy dysfunction and the formation of reactive oxygen and nitrogen species (Rapoport, 2003; Kopeikina et al., 2011).

Neurons begin to die early in AD pathogenesis. Recently, it was shown that microglial phenotypes and their impact on neuron death vary according to disease stage. It will be important to fully understand how this happens and which changes may contribute to neurodegeneration. Astrocytes can also influence neuron death through ApoE expression. Along with their distinct functions in neuroinflammation, there is mounting evidence that microglia and astrocytes cooperate in harmful feedforward loops in AD. The research of Ben Barres’ group showed that cytokine release by microglia may activate astrocytes, leading them to lose part of their normal roles and become toxic to neurons (Liddelow et al., 2017). Two studies in AD models indicate that IL-10 worsens amyloid-related symptoms in mice and also involve microglial IL-10 in increasing astrocytic production of ApoE, which subsequently loops back to decrease microglial attraction for Aβ (Chakrabarty et al., 2015; Guillot-Sestier et al., 2015).

Two pathways of neuronal death in AD have been suggested: one affecting tangle-bearing neurons and resulting in ghost extracellular tangles, and another affecting tangle-free neurons and resulting, at least in part, in apoptosis (Woodhouse et al., 2006). Emerging results from animal models show that inflammation-induced tau hyperphosphorylation and missorting destabilizes the microtubule-actin network and compromises axonal transport by causing microtubule and other organelle aggregation. Disrupted energy metabolism in the axon leads to more tau phosphorylation and impaired axonal transport, resulting in total blockage and axonal leakage, loss of synaptic connections, and activation of microglia and widespread astrogliosis (Krstic and Knuesel, 2013).

Nonetheless, growing evidence from cell and animal studies implicates cell-cell interactions in damaging synapses and neuronal circuits, emphasizing the need for more research to better understand the interplay between cells in the brain and the time course of the alterations.

## 4 Concluding remarks and future perspectives

Healthy brain function is dependent on the integrity of the neural system, which includes the meticulously synchronized work of different cell types. This review covers interactions between the different brain cells (Figure 1) and the respective alterations in AD (Figure 2), which have been linked to a dysregulated functioning of neurovascular and gliovascular units, loss of BBB integrity, excessive neuroinflammation, altered angiogenesis, and neurogenesis. This process results in dendritic loss, synaptic nerve damage, altered neurotransmission, and a generalized hippocampal and cortical degeneration. Here, we examine: 1) how a failing vascular system contributes to disease progression; 2) how neuroinflammation can signal the loss of brain homeostasis; 3) how microglia, astrocytes, endothelial cells, pericytes, oligodendrocytes and neurons, all contribute to a complex cellular phase of the disease; and 4) how initially benign responses can become chronic, culminating in permanent brain dyshomeostasis.

Despite tremendous advances in the discovery of novel treatment options in animal models of AD, there is currently no treatment for this disease in humans, leaving patients with poor options as they faced decades ago.

**FIGURE 3:**
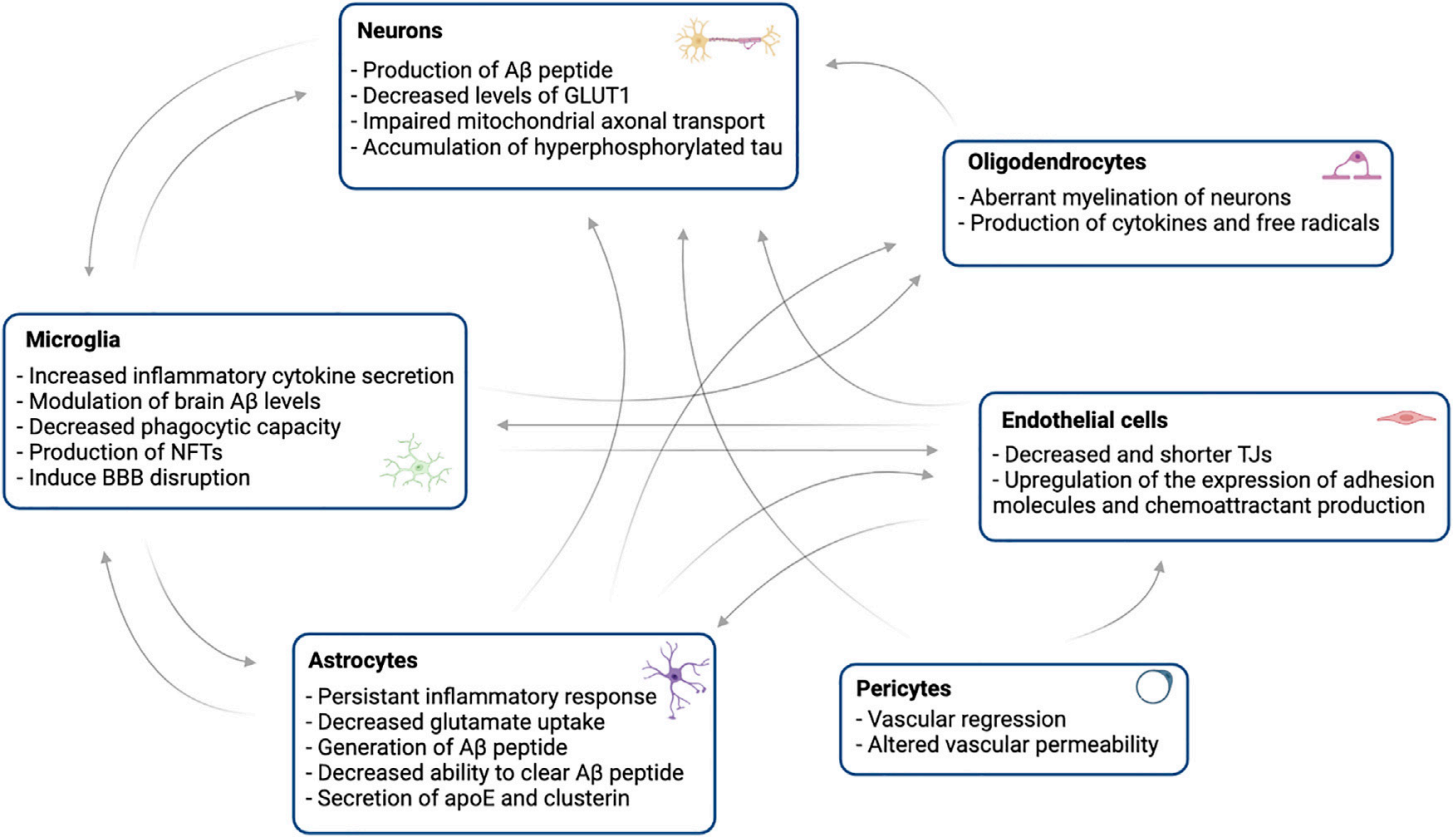
Schematic of the brain cells interactions as early changes in Alzheimer’s disease (AD). To sustain brain function, all cell types in the brain interact in a complex network. Many of these homeostatic functions are compromised in the early stages of AD, and pathways involving different cell types damage synapses and neurons. Impairment in endothelial cells, astrocytic endfeet and pericytes, and the accumulation of Aβ in the vessel walls triggers vascular integrity disruption. These vascular alterations result in impaired vascular protein clearance, hypoperfusion, and BBB disintegration. Amyloid plaques are surrounded by astrocytes and microglia, which initially contribute to remove amyloid protein but become reactive and release cytokines after being exposed to Aβ. In AD, tau accumulates within neurons as neurofibrillary tangles (NFTs), which are linked to glial accumulation, neuronal dysfunction and death. Chronic hypoperfusion compromises white matter integrity by causing oligodendrocyte loss and myelin degradation.

Future research must continue to characterize the sequence of possible cross-talk between distinct cellular signaling pathways (Figure 3), to allow intervention in the first type of brain cell affected, or in the process that leads to a greater amplification of the damage, bringing hope for effective therapies to prevent or treat AD. Overall, new therapies that minimize glial dysfunction and compromised vascular function may play an important role in enhancing neuronal integrity, and triggering increased cognitive function and reducing dementia in AD.
